# Multi-Mode Buckling Analysis of FGM Channel Section Beams

**DOI:** 10.3390/ma13112567

**Published:** 2020-06-04

**Authors:** Monika Zaczynska, Filip Kazmierczyk

**Affiliations:** Department of Strength of Materials, Faculty of Mechanical Engineering, Lodz University of Technology, 90-924 Lodz, Poland; filip.kazmierczyk@dokt.p.lodz.pl

**Keywords:** functionally graded materials, interactive buckling, post-buckling state, thin-walled beams, pure bending

## Abstract

The interactive buckling phenomenon in thin-walled channel section beams is investigated in this paper. This study deals with medium length beams made of the step-variable functionally graded materials (FGM) which consists of aluminum and titanium layers. The interaction of local, primary and secondary global buckling mode and their effect on the load-carrying capacity is discussed. The parametric studies are performed to assess the effect of the thickness of the beam’s component, its length and position of the individual layer on the equilibrium paths. Additionally, the influence of the adhesive layer between materials was analyzed. The problem was solved using the Finite Element Method.

## 1. Introduction

Thin-walled beams are extremely versatile parts used in different branches of industry. Due to the variety of their shapes, they have multiple functions and numerous applications. Elements like guides, girders, or longerons can be made of thin-walled beams. Steel is the most popular material used to form such structures, but nowadays when the optimization of mass becomes more and more important, different materials are used (e.g., composites). GFRP (Glass Fiber Reinforced Polymer) and CFRP (Carbon Fiber Reinforced Polymer) composites are frequently used in the automotive, aerospace, and cycling industry. Due to the orthotropy of such structures, engineers may design the behavior of the detail (i.e., laminate tailoring) based on the working conditions, and therefore parts can be highly optimized. FGM (functionally graded materials) are another type of modern material, which are of interest among researchers due to their huge advantage in providing a smooth change to the mechanical properties through material thickness [1,2]. This gives the possibility to control many parameters, e.g., deformation, dynamic response of FGM structures, and allows the FGM to be tailored for a different application. Thanks to this, FGM structures, e.g., beams, are used in the automotive and aerospace industry as well as in machine elements [3,4,5].

In the worldwide literature, a lot of papers devoted to the problem of stability of FGM structures can be found. Different approaches were applied by various investigators to analyze the loss of the stability phenomenon in FGM members. Analytical, analytical-numerical, and numerical methods are used to examine such structures under various load types. Zhang et al. [6] investigated the thermal buckling of the ceramic–metal structures using the local kriging meshless method based on the local Petrov–Galerkin weak-form formulation and shape functions. Loss of stability under thermal load was also investigated by Babai et al. [7]. The analysis was performed on a functionally graded (FG) porous beam using the third-order shear deformation and physical neutral plane theories. The thermal stability of the FGM plates was analyzed by Do et al. [8,9] with the meshless radial point interpolation method. Zhang et al. [10] investigated the elastoplastic thermal buckling with the symplectic method. Sohn et al. [11] used nine node rectangular elements to investigate the stability of the FGM structure under aero-thermal loads. Moita et al. [12] analyzed FGM and FRP plates under in-plane or thermal load. The instability of FGM plates under thermal load was investigated by Taczala et al. [13]. Prakaash et al. [14] used a shear-deformable finite element method to analyze the FGM plates under in-plane load. In those analyses, the eight-node C0 shear flexible quadrilateral plate elements were applied. An FGM plate subjected to in-plate load was also investigated by Shen [15] who used a semi-analytical approach. Burzynski et al. [16,17] analyzed the stability of FGM structures with the non-linear six-parameter shell theory. Sobhy [18] analyzed the hygrothermal stability of FGM sandwich plates. A new four-variable shear deformation plate theory was introduced in this study. Kumar et al. [19] proposed the new higher-order transverse shear deformation theories (NHSDTs) with five variables to investigate the FGM plate under patch load. Meiche et al. [20] investigated the buckling problem in an FGM sandwich plate using a new hyperbolic shear deformation theory. The problem of loss of stability in FGM beams was investigated in [21,22]. Also, the dynamic buckling in FGM structures is widely investigated by researchers. As an example, the following works can be mentioned: Kiani et al. [23,24,25], Czechowski et al. [26,27,28], Zhang et al. [29,30].

All above presented papers deal with the problem of stability under various load type conditions, such as thermal and mechanical ones. In the world-wise literature, only a few works which are devoted to the problem of the interactive buckling in FGM structures could be found. Kolakowski [31] analyzed the dynamic interactive buckling in trapezoidal FGM beam-columns. Kolakowski and Teter investigated the interactive buckling in thin-walled FGM structures of closed [32] and open [33] cross-sections subjected to the static compression. Kolakowski and Mania [34] analyzed the coupling matrix B effect on the interactive instabilities in FML-FGM (Fiber Metal Laminate–Functionally Graded Material) columns. The interactive buckling in FGM-FML structures was also studied in [35].

It is clear that more studies concerning the problem of the interactive buckling have been devoted to steel structures. As an example, the works of Hung et al. [36], Camotim et al. [37,38,39,40], Niu et al. [41,42] and Kolakowski et al. [43,44] can be mentioned. Camotim et al. [37,38] analyzed the effect of the interaction of the local flexural, distortional and global buckling modes on the structure response. Niu et al. [41,42] investigated the distortional-global interaction buckling observed in results of performed experimental and the numerical tests. The analysis was carried out on channel section steel beams where the elastic-plastic model of the material was assumed. Kolakowski [43] developed a Semi Analytical Method (SAM) based on Koiter’s theory which enables analysis of non-linear instabilities from global to local through their interaction. In [44], SAM was used to analyze the influence of the secondary buckling mode on post-buckling behavior of channel section steel beams. Further investigations are shown in the paper [45], where the influence of the beam length on the interactive buckling was analyzed. The secondary global buckling mode was considered, and the most dangerous interval of beam lengths was defined. The verification of SAM using the finite element method (FEM) was performed for steel [46] and FRP (Fiber Reinforced Polymer) [47] beams for the most dangerous beam lengths interval [45]. In the paper [46], analysis of the interaction of buckling modes on the post-buckling behaviour is presented, and modes with the ratio of the critical load for the primary global to local mode around 0.75 and secondary global to primary one around 10 were considered. In the paper [47], ratios of the considered bifurcation loads were in the interval 0.6–1.4 and 4–12.

The strongest influence of the different buckling mode interaction can be noticed when the ratio of the primary global mode to the local one is in the interval of 0.8–1.2, which has been proved in papers [32,33,34,35,37,38,41,42,43,44,45,46]. It was decided to analyze wider range of ratios and it is 2.6–4.3 for primary global mode to local one and 4–12 for secondary mode to primary one.

The authors of this paper paid special attention to the problem of the interactive buckling in the FGM beams subjected to pure bending. Parametric studies were performed to analyze the influence of the beam wall thickness, the volume percentage of the beam’s components and the location of the aluminum and titanium layers (inside or outside) on the post-buckling behavior. Two various lengths of the beam were considered. Moreover, it was decided to investigate the effect of the adhesive layer present between constituent materials.

## 2. Materials and Methods

**Figure 1:**
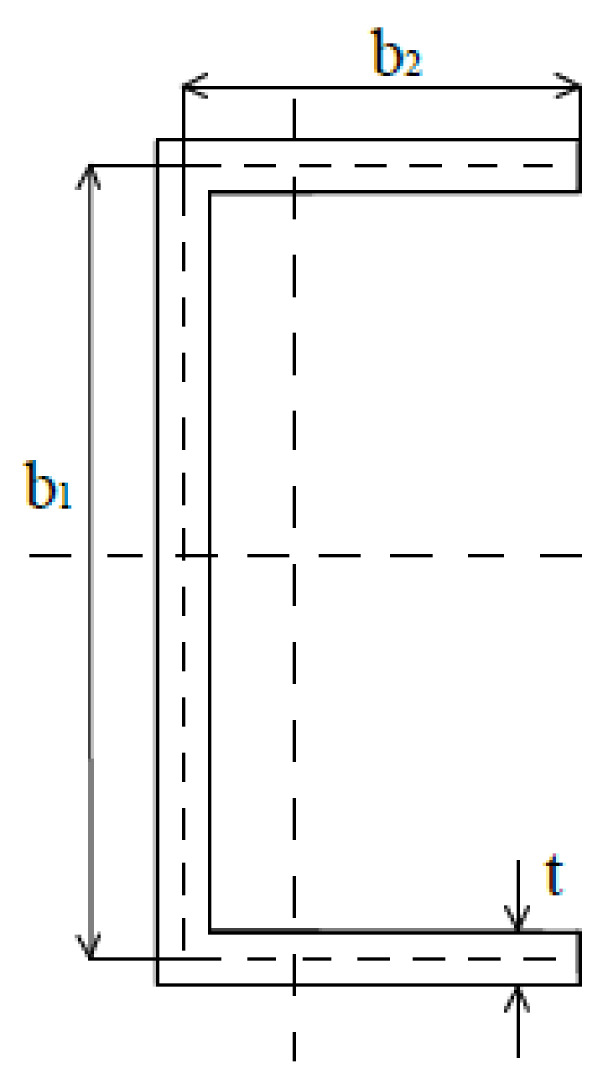
Dimensions of the cross-section.

**Figure 2:**
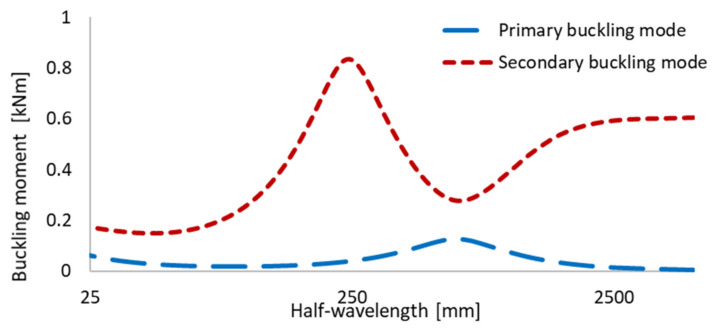
Signature curve of exemplary functionally graded material (FGM) beam.

Numerical analyses were performed using the finite element method (ANSYS APDL software) [48]. Thin-walled channel section beams with the geometry description presented in Figure 1, and their values given in Table 1, were taken into consideration. Beams composed of aluminum Al and titanium Ti sheets with a different thickness of each component (t_Al_ and t_Ti_) were analyzed. The influence of the adhesive layer between aluminum and titanium sheets was taken into account. Two different total walls thickness t = 1 mm (or with adhesive layer t = 1.1 mm) and t = 2 mm (or with adhesive layer t = 2.1 mm) as well as two variants A (aluminum sheet outside) and B (aluminum sheet inside) were studied. The lengths of the beams were chosen, based on the signature curve (Figure 2), to obtain the highest influence of the secondary global buckling mode.

The signature curve presents the critical buckling load in the function of half-wavelength in the longitudinal direction of the beam. Figure 2 presents the curve for the exemplary FGM beam. The lower curve refers to the lowest values of the buckling mode, while the upper one corresponds to the similar beams’ behavior but under higher values of the critical load. Kolakowski and Jankowski [44,45] found out that the highest influence of the secondary buckling mode is observed for the medium-length beams where the range of half-wave length is located on the decreasing upper signature curve (denoted as secondary buckling mode in Figure 2). Thus, based on these works [44,45], the medium-length beams with the length L = 300 mm and L = 400 mm were chosen. Table 2 summarizes the analyzed samples and Table 3 shows mechanical properties of FGM beam components. It was assumed that the materials, from which the beams are made, obey Hooke‘s law. Many samples were taken into consideration, which differs in:

- The location of the aluminum and titanium layer (variant A and B),
- The thickness of aluminium and titanium sheet (t_Al_/t_Ti_ = 1/3, t_Al_/t_Ti_ = 1 and t_Al_/t_Ti_ = 3),
- The existence of adhesive layer t_Ad_ (samples with and without an adhesive layer—t_Ad_ = 0 or t_Ad_ = 0.1 mm),
- The length of the beam (L = 300 mm and L = 400 mm).

**Figure 3:**
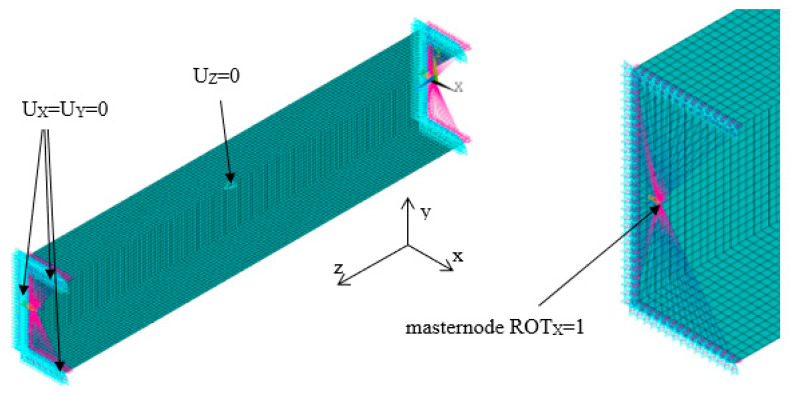
Discretized numerical model with applied boundary conditions.

**Figure 4:**
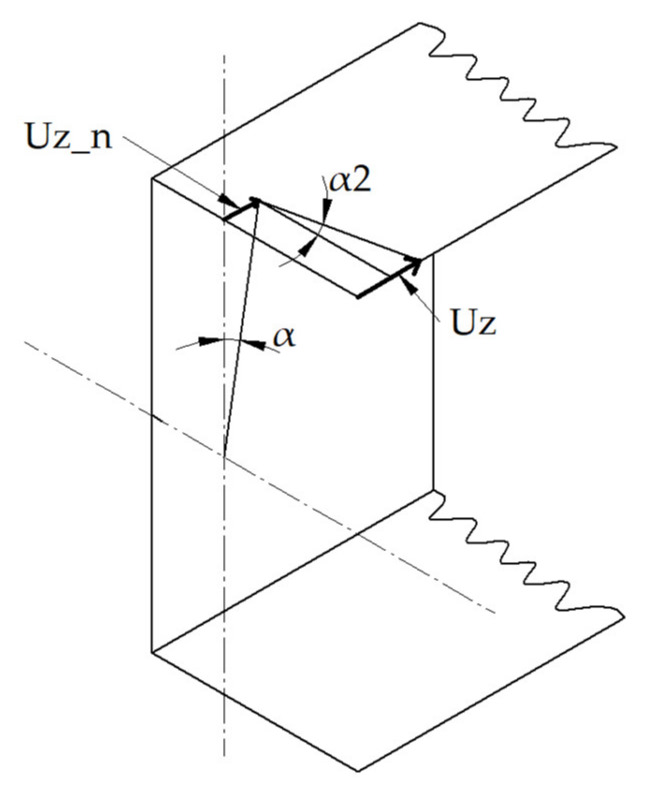
The scheme of the angles of rotation α and α_2_ at the beam’s end.

The numerical models were created using the structural SHELL 181 element. This element, with six degrees of freedom at each node, is described with first order shear deformation theory (Mindlin–Reissner shell theory). This finite element might be used in thin-walled structures to model the multi-layer structures [44]. The size of the finite element is equal to 3 mm in uniform mesh and it was determined based on the experience gained from the previous studies [46,47]. Boundary conditions were applied in the manner to ensure the pure bending with respect to major centroidal axis (Figure 3). Displacements in the vertical and transverse directions were blocked in the nodes located at both beam ends. The displacement in the longitudinal direction was set to zero in the point lying in mid-length of the beam and mid-width of the web. The load was introduced in the form of the angle of rotation in the bending plane. It was applied in the ‘master node’ which is located in the center of gravity of the cross-section and transferred to all nodes at both ends via RBE 2 elements via RBE 2 elements. The angle of rotation α was determined directly from the applied load while bending moment *M* was determined as the reaction (moment) in the ‘master node’ [46,47]. Additionally, the angle of rotation around the Y-axis, denoted as α_2,_ was tracked. The analyzed angles α and α_2_ at the beam’s end are presented in Figure 4. The angle α_2_ was calculated using the following formula:

(1) $$\alpha_{2}=\mathrm{arctg}\frac{U_{z}-U_{z\_n}}{x_{\max}}$$

where:

U_z_—Displacement of the point located on the free corner of compressed flange,

U_z_n_—Displacement in the z-direction of the point located on the compressed flange and the central vertical axis,

x_max_—Maximal distance from the central axis to the outer layer in the x-direction.

## 3. Results

In this section the results from performed numerical analysis are shown. Linear and non-linear buckling analysis were considered in order to obtain the full spectrum of the beam’s behavior. Critical buckling moments as well as the angle of rotation and equilibrium paths were determined.

### 3.1. Linear Buckling Analysis

Linear buckling analysis was performed using the Block Lanczos method to determine the buckling moments and corresponding angles of rotation for following buckling modes:

- Local buckling mode (m > 1, where m is the number of half-waves in the longitudinal direction)
- Primary global-distortional buckling mode (m = 1)
- Secondary global-distortional buckling mode (m = 1 s)

**Figure 5:**
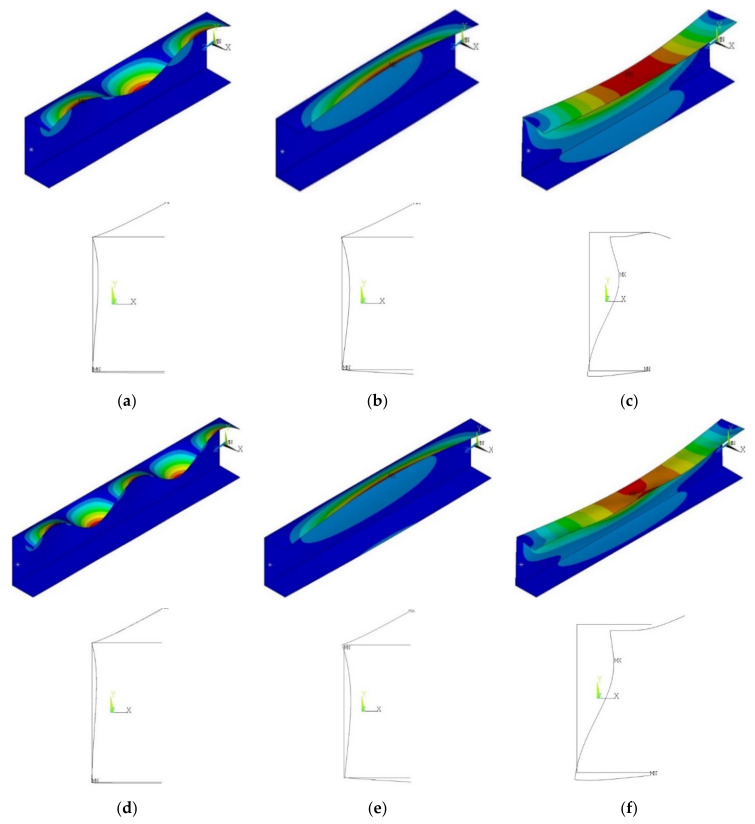
Finite element method (FEM) buckling modes: (**a**) m = 3—local buckling mode for L = 300 mm; (**b**) m = 1 global distortional-lateral buckling mode for L = 300 mm; (**c**) m = 1 s secondary global distortional-lateral buckling mode for L = 300 mm; (**d**) m = 5—local buckling mode for L = 400 mm; (**e**) m = 1 global distortional-lateral buckling mode for L = 400 mm; (**f**) m = 1 s secondary global distortional-lateral buckling mode for L = 400 mm.

Considered buckling modes and the corresponding beam deflections are presented in Figure 5 while the buckling moments and corresponding angles of rotation are listed in Table 4. For beams with the length of 300 mm, the lowest buckling mode has the local character with three half-waves in the longitudinal direction, while for the beams with the length of 400 mm the lowest buckling mode was also the local mode, but with five half-waves in the longitudinal direction. The analysis of the deflection of the beam cross-section depicted that the secondary buckling mode for a 400 mm-length beam (Figure 5f) presents little greater flexural-distortional character compared to beam with L = 300 mm (Figure 5c).

Comparing the bifurcation loads for cases A and B (aluminium layer inside or outside), it could be observed that the localization of the FGM beam component has a negligible effect. Another conclusion could be made in the case of the adhesive layer. Implementation of the adhesive layer to the numerical models leads to the increase of the buckling loads (about 30%). Local bifurcation load is over 2.5 times smaller in comparison to primary global one (i.e., M_cr_ (m = 1)/M_cr_ (m > 1) > 2.6 and M_cr_ (m = 1 s)/M_cr_ (m = 1) > 4.7).

### 3.2. Non-Linear Buckling Analysis

The non-linear buckling analysis with initial geometric imperfection was performed using the Newton–Raphson algorithm. The initial geometric imperfections were applied to the model with the shape corresponding to the considered buckling modes. The magnitude of the imperfection was assumed as 100% of wall thickness for primary and secondary global-buckling mode and 10% for a local one. Positive and negative imperfections were analyzed and the worst case of the sign (for which the lowest critical buckling moment was obtained) was chosen. Graphs of equilibrium paths were tracked for all analyzed samples. They show the ratio of buckling moment to the critical buckling moment (M/M_cr_) in the function of angle of rotation to the critical one (α/α_cr_).

**Figure 6:**
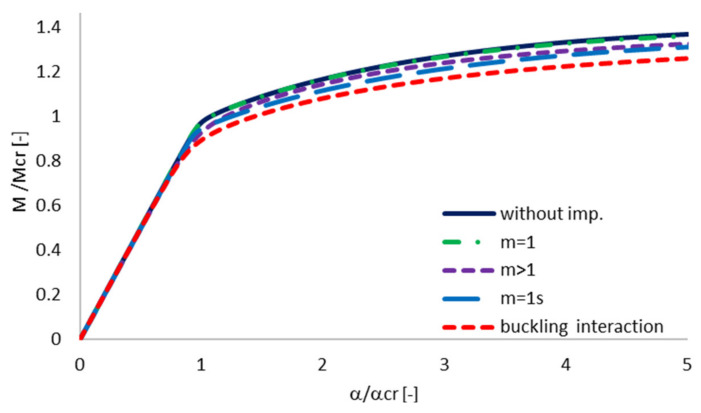
The effect of different initial geometric imperfection on equilibrium path for 4A_50 beam.

As the buckling interaction is analyzed, it is important to show the effect of each individual buckling mode as well as the interaction of the considered buckling modes on the equilibrium paths. The influence of different initial geometric imperfection on post-buckling equilibrium path is presented for one exemplary beam (beam 4A_50) in Figure 6. It was observed that initial imperfections affect post-buckling regime. In the pre-buckling range, all curves are almost coincident. Analyzing the one-mode approach, the highest effect on the structure response is observed when the geometric imperfections that corresponded to the secondary global distortional buckling mode are applied. Comparing all curves presented in Figure 6, the highest decrease of post-buckling stiffness and load-carrying capacity is obtained for multi-mode interaction.

### 3.3. The Effect of Aluminium and Titanium Thickness and Position

**Figure 7:**
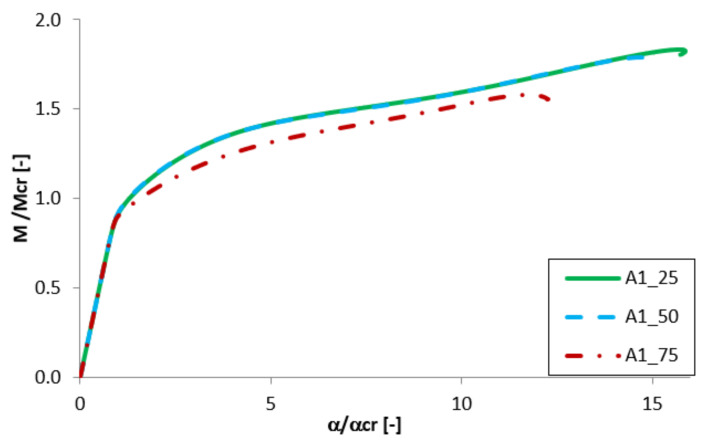
Comparison of equilibrium paths for A beam with a total thickness of 1 mm.

**Figure 8:**
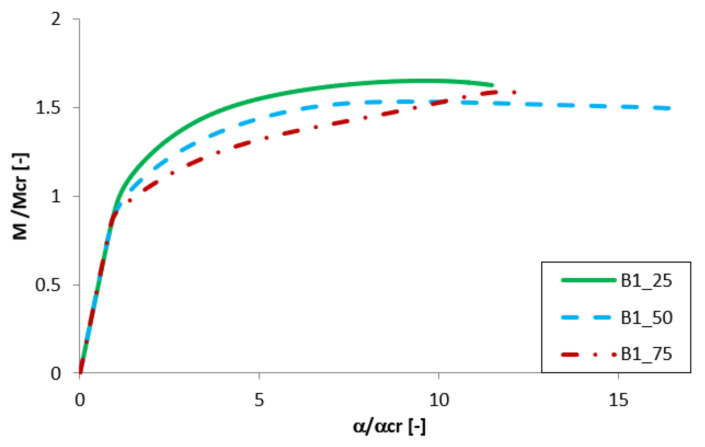
Comparison of equilibrium paths for B beam with a total thickness of 1 mm.

**Figure 9:**
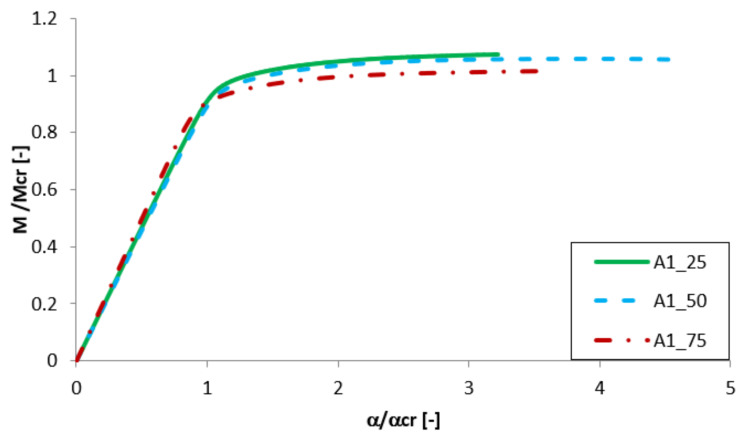
Comparison of equilibrium paths for A beam with a total thickness of 2 mm.

**Figure 10:**
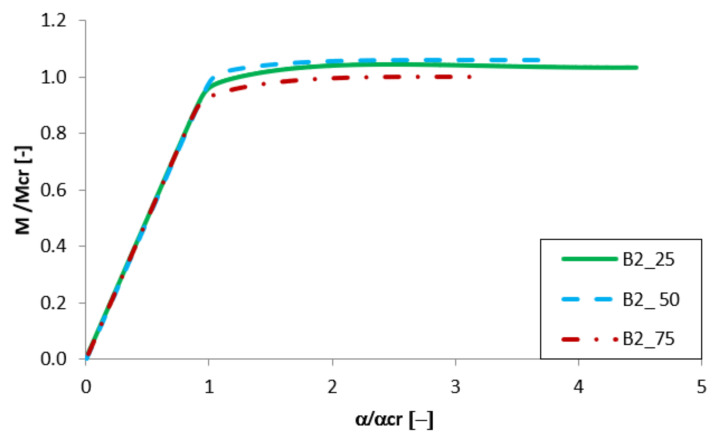
Comparison of equilibrium paths for B beam with a total thickness of 2 mm.

Equilibrium paths in the dimensionless form are presented in Figure 7, Figure 8, Figure 9 and Figure 10. The effect of the aluminium and titanium layer thickness for the beam with a total thickness of 1 mm is presented in Figure 7 and Figure 8, while for the cases with a total thickness of 2 mm in Figure 9 and Figure 10. Comparing both variants (A and B) for t = 1 mm, a greater influence of geometric imperfections on post-critical paths was observed for variant B (Figure 7 and Figure 8). The results shown in Figure 7, indicate the same stiffness in post-buckling range for beams A_25 and A_50. A beam with 75% of titanium presents lower stiffness. For variant A, the following tendency is observed—With the increase of the aluminium layer thickness, the non-dimensional load-carrying capacity decreases. This tendency is also valid for a beam with a thickness of 2 mm. However, for such beams (t = 2 mm) the effect of the aluminium layer position (outside—A or inside—B) is negligible.

Both variants A and B with assumed imperfections have ‘raising’ post-critical paths M/M_cr_ > 1 for the thickness of 1 mm, while for the thickness of 2 mm the load-carrying capacity practically does not exceed the critical load (i.e., M/M_cr_ ≈ 1). It proves that the higher internal forces occur in the beams with a thickness of 2 mm than for those with t = 1 mm [47].

### 3.4. The Effect of the Adhesive Layer

**Figure 11:**
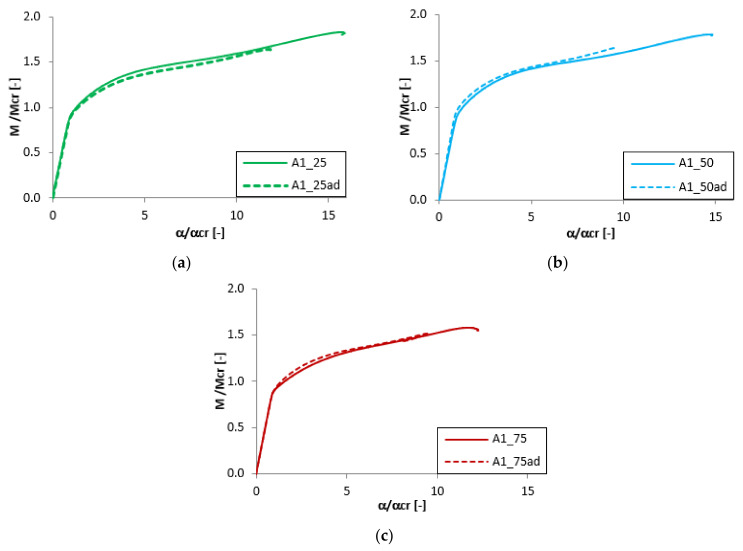
The effect of the adhesive layer for A beam with 1 mm thickness: (**a**) t_Ti_ = 0.25 mm; (**b**) t_Ti_ = 0.50 mm; (**c**) t_Ti_ = 0.25 mm.

**Figure 12:**
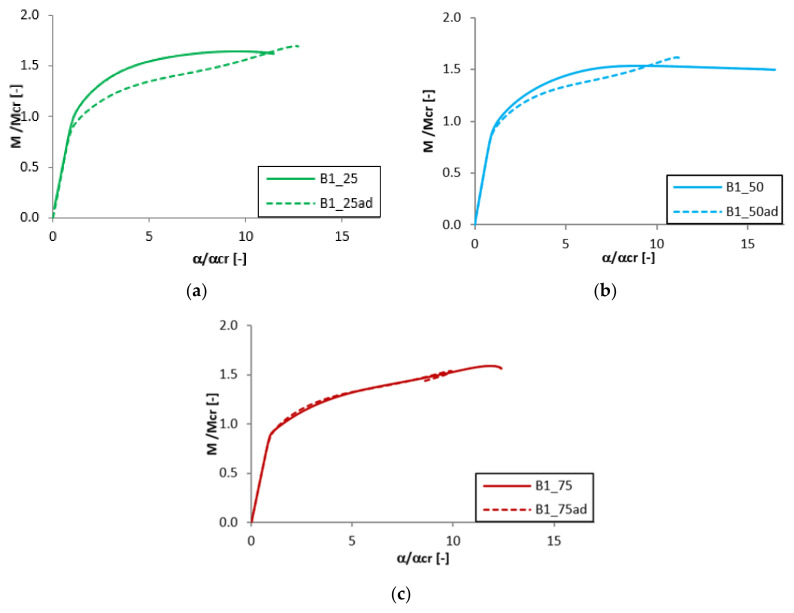
The effect of the adhesive layer for B beam with 1 mm thickness: (**a**) t_Ti_ = 0.25 mm; (**b**) t_Ti_ = 0.50 mm; (**c**) t_T*i*_ = 0.25 mm.

**Figure 13:**
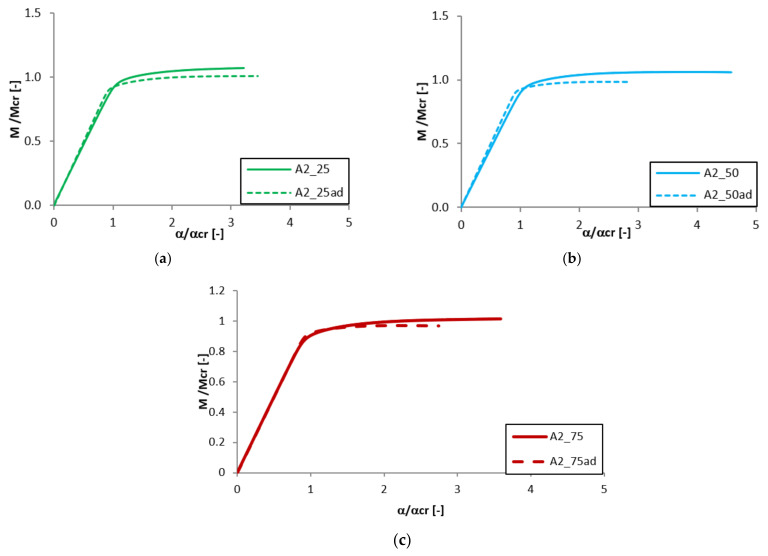
The effect of the adhesive layer for A beam with 2 mm thickness: (**a**) t_Ti_ = 0.50 mm; (**b**) t_Ti_ = 1.00 mm; (**c**) t_Ti_ = 1.50 mm.

**Figure 14:**
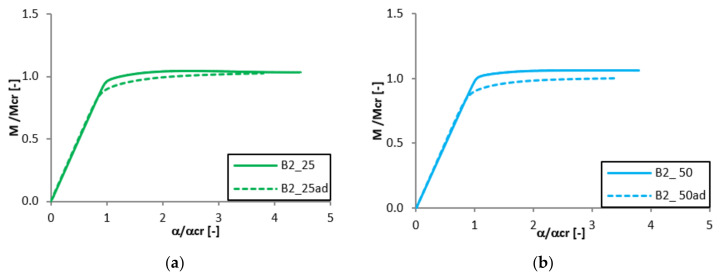

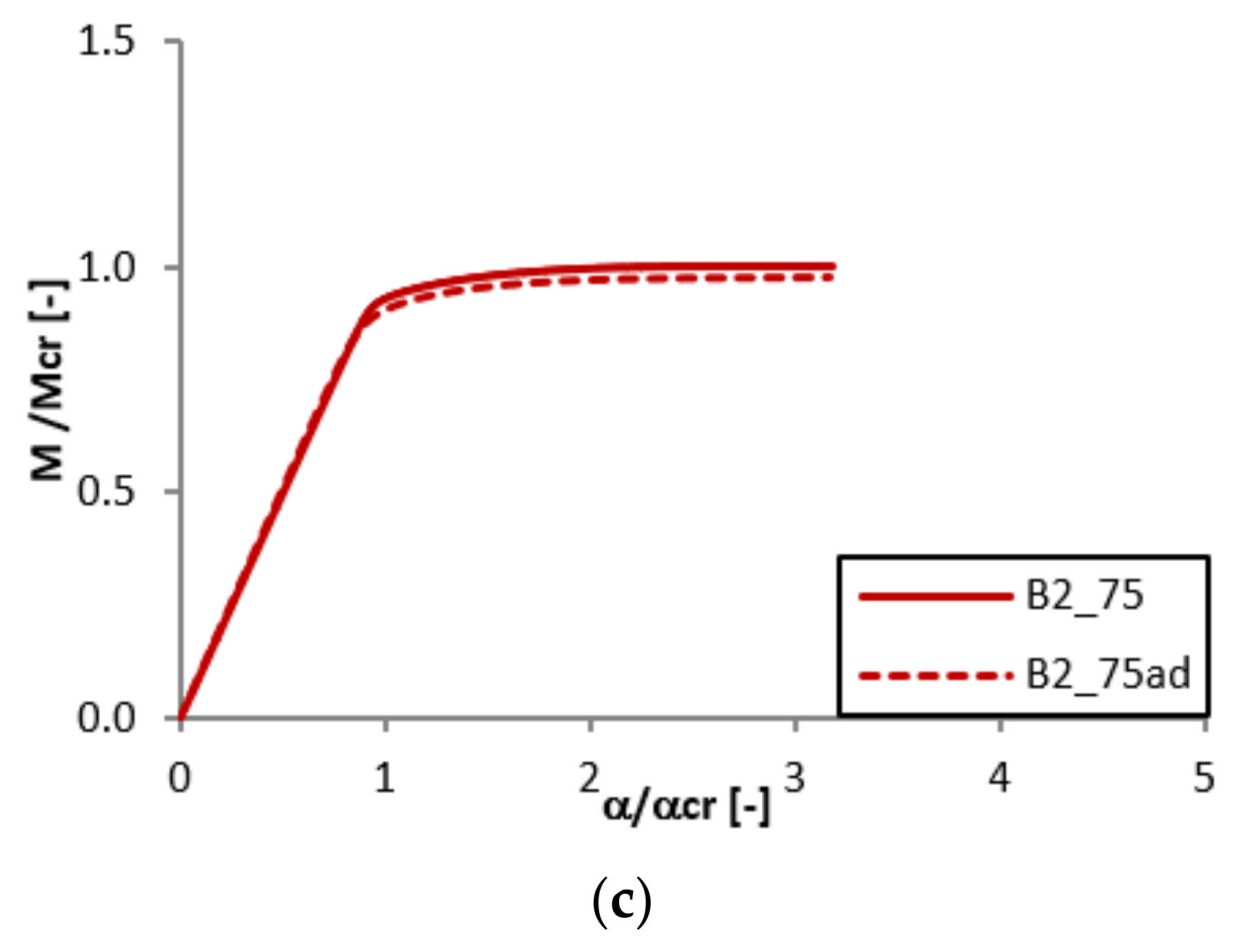
The effect of the adhesive layer for B beam with 2 mm thickness: (**a**) t_Ti_ = 0.50 mm; (**b**) t_Ti_ = 1.00 mm; (**c**) t_Ti_ = 1.50 mm.

The analysis of the adhesive layer influence on the equilibrium paths is presented in Figure 11, Figure 12, Figure 13 and Figure 14. For almost all considered cases (except the beam B1_25 and B1_50) the adhesive layer leads to the decrease of the dimensionless load-carrying FGM beam capacity. Simultaneously, such a tendency of the equilibrium paths (mainly in the post-buckling range) remained unchanged. The only exceptions were the samples B1_25 and B1_50, where the addition of the adhesive layer led to the increase of the load-carrying capacity and the change of the post-critical behavior (Figure 12). For the beam A1_25 and B1_75ad (Figure 11 and Figure 12), a rapid decrease of the dimensionless bending moment and angle of rotation in the post-critical range was observed, and this phenomenon was deeply investigated in references [46,47,50].

### 3.5. The Effect of the Beam’s Length

**Figure 15:**
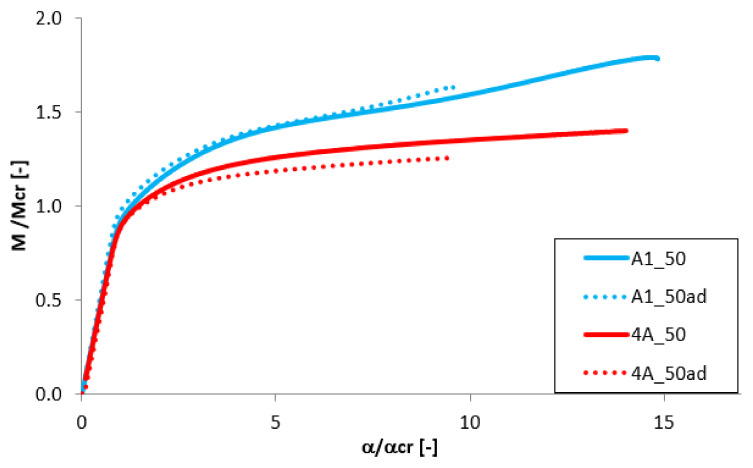
The effect of the beam length for variant A.

**Figure 16:**
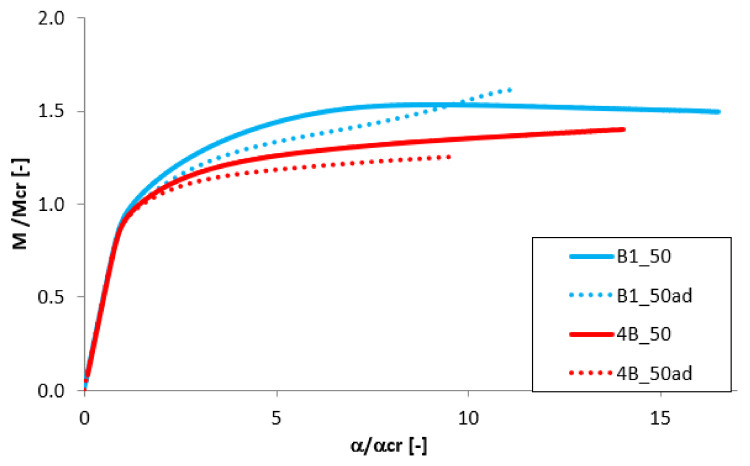
The effect of the beam length for variant B.

To analyze the effect of the beam’s length on the post-critical equilibrium paths, the results for beams with length of 300 mm and 400 mm and of equal thickness of individual layer t_Al_ = t_Ti_ = 0.5 mm were compared. The results presented in Figure 15 and Figure 16 indicate the high differences in the post-buckling response. For shorter beam with L = 300 mm a slight increase of the bending moment is observed after the loss of stability (Figure 15), while for the longer beam the load increases slightly. Simultaneously, almost 40% drop of the dimensionless load-carrying capacity is observed for the beam with 400 mm length. For the variant B the difference in the maximum load is significantly smaller. However, also in this case, the change of the post-buckling equilibrium path occurs.

The presented tendency, in the results for analyzed samples could be explained by the difference in the shape of the buckling modes. As presented in Figure 5, the secondary distortional buckling mode for the beam with the length L = 400 mm presents a higher distortional effect (the entire displacement of the beam cross-section).

In Table 5, the dimensionless load-carrying capacity is compared for all cases. Interactive buckling results of FGM beams with 1 mm of a total wall thickness indicate the significant increase of the load-carrying capacity (M_max_/M_cr_ > 1.5 for L = 300 mm and M_max_/M_cr_ > 1.2 for L = 400 mm), while for the beams with a total wall thickness of 2 mm the ratio of M_max_/M_cr_ it takes place at around 1. The dimensionless load-carrying capacity remains almost unchanged with the different position of the aluminium layer in the FGM structure (cases A and B). An analogous tendency was obtained for the buckling load M_cr_ (Table 4).

In the presented paper authors have analyzed the impact of the modes for which the ratio of primary global mode to local one is higher than 2.6 and secondary global mode to primary one is higher than 4. Obtained results are in the good agreement with conclusions formulated in papers [44,45,46,50] about the impact of the secondary global buckling mode on the post-bifurcation equilibrium paths and the load-carrying capacity.

### 3.6. The Angle of Rotation at the Beam Ends

**Figure 17:**
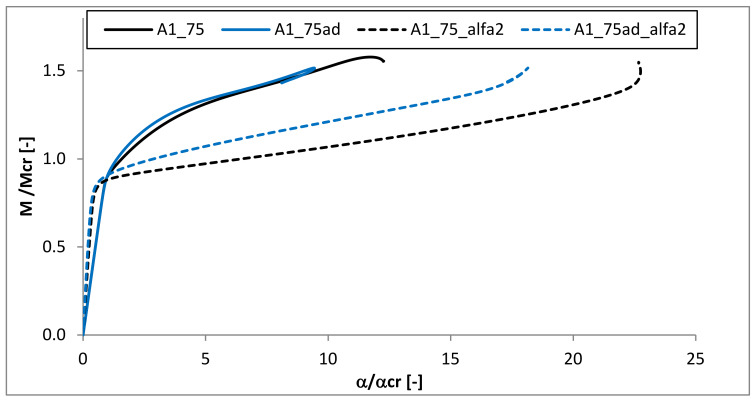
The comparison of the angle of rotation around two axes at the beam end.

For the one exemplary case (A1_75), the comparison of the angles of rotation about two axes at the beam ends is presented in Figure 17. The angle α (angle in the plane of bending—about the major centroidal axis) is presented by solid lines, while the angle angle α_2_ in the plane YZ plane—about minor centroidal axis is marked with dashed ones. The analysis was performed for two samples which differed in the existence of the adhesive layer (A1_75 and A1_75ad). It could be observed, that for both beams, with and without adhesive layers, the angle α was about two times higher than α_2_ in the pre-buckling range. However, just after the critical point, the angle α_2_ increased rapidly. For the ratio M/M_cr_ = 1.2 the angle α_2_ was almost threefold (for A1_75ad) and fivefold (for A1_75) higher than the angle α. This proves the distortional character of the structure buckling. A similar tendency was also observed in [46].

## 4. Conclusions

Numerical investigations of the influence of the geometrical and material parameters on buckling and post-buckling behaviour of step-variable FGM medium-length beams were performed. Firstly, linear buckling analysis was carried out using ANSYS^®^ 18.2 software. Subsequently, the non-linear analysis with the complex initial geometric imperfection was performed. It was observed that the thickness of the FGM components has an effect on the post-buckling equilibrium path, mainly for the beams with the total wall thickness of 1 mm. Implementation of the adhesive layer to the numerical models leads to similar buckling loads and to a slight decrease of the dimensionless load-carrying capacity. Comparing the results obtained for the beams of two lengths, it could be observed that for the longer beam the post-buckling equilibrium path was flatter. Additionally, the change of angle of rotation was analyzed. Rapid growth of the α_2_ angle in the post-buckling range was observed. The crucial influence of the secondary global buckling mode on the post-bifurcation beams’ behaviour under pure bending was confirmed.

## Figures and Tables

**Table 1:** Characteristic parameters of the considered beams.

Total Wall Thickness—t (mm)	Width of the Web—b_1_ (mm)	Width of the Flange—b_2_ (mm)	Length of the Beam—L (mm)
1–2.1	80	40	300; 400

**Table 2:** Lists of the analyzed samples.

L (mm)	Name	Case	Thickness (mm)			Name	Case	Thickness (mm)		
			t_Al_	t_Ti_	t_Ad_			t_Al_	t_Ti_	t_Ad_
**300**	A1_75	A	0.25	0.75	0.00	B1_75	B	0.25	0.75	0.00
	A1_75ad	A	0.25	0.75	0.10	B1_75ad	B	0.25	0.75	0.10
	A1_50	A	0.50	0.50	0.00	B1_50	B	0.50	0.50	0.00
	A1_50ad	A	0.50	0.50	0.10	B1_50ad	B	0.50	0.50	0.10
	A1_25	A	0.75	0.25	0.00	B1_25	B	0.75	0.25	0.00
	A1_25ad	A	0.75	0.25	0.10	B1_25ad	B	0.75	0.25	0.10
	A2_75	A	1.50	0.50	0.00	B2_75	B	1.50	0.50	0.00
	A2_75ad	A	1.50	0.50	0.10	B2_75ad	B	1.50	0.50	0.10
	A2_50	A	1.00	1.00	0.00	B2_50	B	1.00	1.00	0.00
	A2_50ad	A	1.00	1.00	0.10	B2_50ad	B	1.00	1.00	0.10
	A2_25	A	0.50	1.50	0.00	B2_25	B	0.50	1.50	0.00
	A2_25ad	A	0.50	1.50	0.10	B2_25ad	B	0.50	1.50	0.10
**400**	4A_50	A	0.50	0.50	0.00	4B_50	B	0.50	0.50	0.00
	4A_50ad	A	0.50	0.50	0.10	4B_50ad	B	0.50	0.50	0.10

**Table 3:** Mechanical properties of FGM’s beam components.

Material	E (GPa)	*ν*
Titanium Ti-5Al-2.5Sn	107	0.34
Aluminium EN AW-2024 (AlCu4Mg1)	71	0.33
Adhesive Araldite AW 4804 [49]	6.2	0.4

**Table 4:** Comparison of buckling loads.

Name	Mode	M_cr_ (Nm)	α_cr_ (°)	Name	Mode	M_cr_ (Nm)	α_cr_ (°)	M_cr_(A)/M_cr_(B) (-)
A1_75	3	178	0.11	B1_75	3	177	0.11	1.01
	1	502	0.32		1	497	0.31	1.01
	1 s	6516	4.11		1 s	6546	4.12	0.99
A1_75ad	3	226	0.14	B1_75ad	3	225	0.14	1.00
	1	635	0.40		1	631	0.39	1.01
	1 s	6732	4.21		1 s	6774	4.23	0.99
A1_50	3	181	0.10	B1_50	3	179	0.10	1.01
	1	510	0.29		1	505	0.29	1.01
	1 s	7170	4.07		1 s	7241	4.10	0.99
A1_50ad	3	237	0.13	B1_50ad	3	235	0.13	1.01
	1	670	0.38		1	662	0.37	1.01
	1 s	7419	4.18		1 s	7503	4.22	0.99
A1_25	3	188	0.10	B1_25	3	187	0.10	1.01
	1	532	0.27		1	528	0.27	1.01
	1 s	7815	40.39		1 s	7889	4.05	0.99
A1_25ad	3	229	0.12	B1_25ad	3	227	0.12	1.01
	1	647	0.33		1	639	0.33	1.01
	1 s	8053	4.13		1 s	8143	4.16	0.99
A2_75	3	1389	0.43	B2_75	3	1408	0.44	0.83
	1	3726	1.18		1	3666	1.15	0.85
	1 s	14810	4.66		1 s	15008	4.72	0.88
A2_75ad	3	1574	0.49	B2_75ad	3	1556	0.49	1.01
	1	4147	1.30		1	4089	1.30	1.01
	1 s	15083	4.74		1 s	15257	4.74	0.99
A2_50	3	1410	0.36	B2_50	3	1433	0.41	0.98
	1	3821	1.09		1	3748	1.06	1.02
	1 s	16,226	4.61		1 s	16,519	4.67	0.98
A2_50ad	3	1629	0.46	B2_50ad	3	1600	0.46	1.02
	1	4332	1.23		1	4242	1.23	1.02
	1 s	16,566	4.69		1 s	16,851	4.69	0.98
A2_25	3	1474	0.38	B2_25	3	1491	0.38	0.99
	1	3991	1.03		1	3939	1.01	1.01
	1 s	17,873	4.60		1 s	18,067	4.64	0.99
A2_25ad	3	1638	0.42	B2_25ad	3	1612	0.42	1.02
	1	4370	1.12		1	4293	1.12	1.02
	1 s	18060	4.64		1 s	18330	4.64	0.99
4A1_50	5	181	0.14	4B1_50	5	179	0.14	1.01
	1	784	0.59		1	776	0.59	1.01
	1 s	4643	3.51		1 s	4684	3.53	0.99
4A1_50ad	5	237	0.18	4B1_50ad	5	235	0.18	1.01
	1	1017	0.76		1	1004	0.75	1.01
	1 s	4786	3.60		1 s	4836	3.62	0.99

**Table 5:** Comparison of load-carrying capacity.

Name	M_max_/M_cr_	Name	M_max_/M_cr_
A1_75	1.578	B1_75	1.589
A1_75ad	1.516	B1_75ad	1.539
A1_50	1.788	B1_50	1.532
A1_50ad	1.637	B1_50ad	1.613
A1_25	1.832	B1_25	1.649
A1_25ad	1.646	B1_25ad	1.695
A2_75	1.014	B2_75	1.002
A2_75ad	0.971	B2_75ad	0.977
A2_50	1.060	B2_50	1.062
A2_50ad	0.986	B2_50ad	0.99
A2_25	1.074	B2_25	1.045
A2_25ad	1.016	B2_25ad	1.028
4A1_50	1.399	4B1_50	1.437
4A1_50ad	1.256	4B1_50ad	1.267
